# Treatment of osteoporosis in an older home care population

**DOI:** 10.1186/1471-2474-6-7

**Published:** 2005-02-11

**Authors:** Shelly A Vik, Colleen J Maxwell, David A Hanley

**Affiliations:** 1 Department of Community Health Sciences University of Calgary, Calgary AB, Canada; 2 Medicine, University of Calgary, Calgary AB, Canada; 3 Institute of Health Economics, Edmonton AB, Canada

## Abstract

**Background:**

Previous research indicates that many patients with fractures indicative of underlying osteoporosis are not receiving appropriate diagnostic follow-up and therapy. We assessed osteoporosis treatment coverage in older home care clients with a diagnosis of osteoporosis and/or prevalent fracture.

**Methods:**

Subjects included 330 home care clients, aged 65+, participating in a longitudinal study of medication adherence and health-related outcomes. Data on clients' demographic, health and functional status and service utilization patterns were collected using the Minimum Data Set for Home Care (MDS-HC). A medication review included prescribed and over-the-counter medications taken in the past 7 days. Criteria for indications for osteoporosis therapy included diagnosis of osteoporosis or a recent fracture. Coverage for treatment was examined for anti-osteoporotic therapies approved for use in 2000.

**Results:**

Of the 330 home care clients, 78 (24%) had a diagnosis of osteoporosis (n = 47) and/or had sustained a recent fracture (n = 34). Drug data were available for 77/78 subjects. Among the subjects with osteoporosis or a recent fracture, 45.5% were receiving treatment for osteoporosis; 14% were receiving only calcium and vitamin D, and an additional 31% were receiving drug therapy (bisphosphonate or hormone replacement therapy). The remaining 54.5% of subjects were not receiving any approved osteoporosis therapy.

**Conclusions:**

The high prevalence of undertreatment among a population of older adults with relatively high access to health care services raises concern regarding the adequacy of diagnosis and treatment of osteoporosis in the community.

## Background

Osteoporosis is defined as ' . . .a skeletal disorder characterized by compromised bone strength predisposing a person to an increased risk of fracture. Bone strength reflects the integration of two main features; bone density and bone quality' [1]. The presence of a vertebral or other fragility fracture is a strong predictor of risk of future fracture, and is a major indicator of the presence of osteoporosis [2-4]. A fragility fracture is defined as a low trauma fracture (eg. a fall from a standing height or less) [3]. Many studies indicate that fewer than 30% of patients with fragility fractures are being treated for osteoporosis. Rates of treatment appear particularly low following hospital discharge (<10%) [5-7], suggesting that treatment recommendations are not being made to family physicians. Assessment of treatment rates 6 months to 2 years after fracture are somewhat higher (in the 20–38% range) [8-13]. Kiebzak et al. followed patients for 1–5 years after hip fracture. At follow-up, they found that 71% of women were receiving treatment, but the proportion of men receiving therapy remained relatively low [14]. Four Canadian studies of osteoporosis treatment following fragility fracture were identified. Two studies examined osteoporosis treatment among hip fracture patients after hospital discharge, and in both, treatment rates were relatively low (< 10%) [6,15]. In Ontario, Hajcsar et al. interviewed patients one year after fragility fracture, and found that the use of approved pharmaceutical agents such as bisphosphonates and hormone replacement therapy (HRT) remained low (7.4% and 16%, respectively) [13]. Khan et al. reported considerably higher treatment rates among patients after wrist fracture, with approximately 38% of patients receiving either a bisphosphonate or HRT at follow-up [9].

The purpose of this study was to examine osteoporosis treatment among older home care clients with a diagnosis of osteoporosis and/or prevalent fracture. As this population has relatively frequent contact with health care providers, we expected that osteoporosis treatment rates should be higher than previously reported.

## Methods

Participants were 330 older home care clients participating in a longitudinal study of medication adherence and related health outcomes. Details of the primary study can be found elsewhere [16]. Briefly, between April and June of 2000, 585 home care clients residing in two southern Alberta health regions were identified by random samples stratified by rural/urban residence. Rural clients represented those living on a farm, acreage or in a village or town (with a population less than 10,000) and residing greater than 35 km from a major urban centre (cities of Calgary or Lethbridge). Inclusion criteria were: currently receiving home care services under the jurisdiction of their respective health region, age 65 or greater and provision of informed consent from either the subject or a legal guardian. Of the original random sample of 585, 10 subjects had died and 10 had moved or were unavailable at the time of study recruitment. Further exclusions included subjects who were in hospital, transferred to long term or palliative care, or mentally incompetent without a legal guardian (n = 40), who required a translator (n = 6) or who posed a safety concern for the study nurses (n = 7). Of the remaining 512 subjects, 46 (9.0%) refused to participate and 136 (26.6%) were not contacted after the predetermined sample size (based on estimation of differences in adherence rates between rural and urban subjects) [16] had been achieved.

Data on demographics, health and functional status and service utilization were collected with a standardized assessment tool, the Minimum Data Set for Home Care (MDS-HC) [17,18], and several supplemental questions regarding medication use, smoking and tobacco use. Information on therapeutic substances was recorded from container labels for all substances used in the past 7 days. Therapeutic substances included prescribed, over-the-counter and complementary or alternative products. All drug data were entered into a database using the Anatomical Therapeutic Chemical classification system (ATC codes). Assessment of medication adherence was based on self-report data [16]. Four study nurses, trained in the administration of the MDS-HC and medication assessment, collected data during in-home interviews lasting approximately 1.5 hours. This study received ethics approval from the Health Research Ethics Board of the University of Calgary and the Ethics Review Committee of the Chinook Health Region.

Fracture prevalence and diagnosis of osteoporosis were determined from the disease diagnoses section of the MDS-HC (Section J). The MDS-HC includes specific categories for charting of: 'hip fracture', 'other fracture', and 'osteoporosis'. Prevalent fractures were defined as those that had resulted in a hospitalization (in last 90 days), currently required treatment and/or symptom management or were being monitored by a home care professional. Coverage for treatment was examined for anti-osteoporotic therapies approved for use in 2000 [19]. These included calcium with vitamin D, and the following pharmaceutical agents: etidronate, alendronate, hormone replacement therapy, raloxifene and calcitonin.

Prevalence estimates of treatment for osteoporosis among subjects with a prevalent fracture and/or diagnosis of osteoporosis were reported. Descriptive bivariate comparisons were also conducted to examine potential variations between osteoporotic subjects who received treatment and those who did not. Fisher's exact test was reported for the bivariate comparisons. Due to the limited number of subjects with osteoporosis or fracture, multivariable analyses were not feasible.

## Results

Demographic and health status variables are summarized in Table 1. The mean age of the total sample (n = 330) was 82 years (standard deviation = 7.8, range 65–101) and most clients were female (78.5%) and not married (70.6%). Almost half (41.8%) had completed at least high school education and a substantial proportion of subjects lived in a seniors' lodge (36.7%). A total of 78 subjects (23.6%) had at least one potential indication for osteoporosis treatment. The demographic characteristics of these subjects were similar to the sample as a whole (Table 1), with the exception that this group included a greater proportion of women (93.6%). Demographic data were only available for a sub-group of all non-respondents. Analyses of this sub-sample showed no significant differences between non-respondents and respondents in relation to age and sex.

**Table 1:** Summary of demographic characteristics and health status among older home care clients.

**Variable**	**Total Study Population N = 330 % (n)**	**Subjects with diagnosis of osteoporosis or fracture N = 78 % (n)**
**Demographics**		
Age		
Mean (SD)	82 (7.8)	83 (8.3)
(≥ 85)	43.3 (143)	42.3 (33)
Female	78.5 (259)	93.6 (73)
Not married	70.6 (233)	79.5 (62)
Education (≥ High School)	41.8 (138)	44.9 (35)
Living arrangements		
Seniors' Lodge*	36.7 (121)	34.6 (27)
**Health Status**		
Cognitive impairment		
(CPS** score >1)	23.3 (77)	24.4 (19)
# Comorbid conditions (>2)	71.8 (237)	71.8 (237)

* *Versus *private home

** MDS-HC Cognitive Performance Scale

The prevalence of osteoporosis diagnoses and fractures are summarized in Table 2. Forty-seven subjects (14.2%) had a diagnosis of osteoporosis, three of whom had at least one prevalent fracture. Prevalence of at least one fracture without diagnosis of osteoporosis was recorded for 31 (9.4%) subjects; 13 hip fractures (1 with 'other fracture'), and 18 with 'other' fractures. One client had only unlabelled medication bottles, thus medication data were available for 77 of these subjects. Approximately 50% of subjects with a diagnosis of osteoporosis or prevalent hip fracture received treatment, compared to only 28% of subjects with 'other' fractures (p = 0.11).

**Table 2:** Indications for treatment of osteoporosis among older home care clients.

	**Diagnoses N = 330**	Receiving treatment* N = 77
**Indication**	**% (n)**	**% (n)**
Diagnosis of osteoporosis**	14.2 (47)	50.0 (23/46^†^)
		
Hip fracture^‡^	3.9 (13)	53.8 (7/13)
'Other' fracture^??^	5.5 (18)	27.8 (5/18)
		
**Total**	23.6 (78)	45.5 (35/77)

* Treatments included: calcium with vitamin D and/or drug therapy (bisphosphonate or hormone replacement therapy).

** Three subjects also had at least one fracture.

^† ^Medications unlabelled for one subject.

^‡ ^One subject also had 'other fracture'

?? Type of fracture was available for 10/18 subjects: 3 wrist, 1 humerus, 2 ankle, 2 vertebral, 1 shoulder and 1 rib fracture.

**Figure 1:**
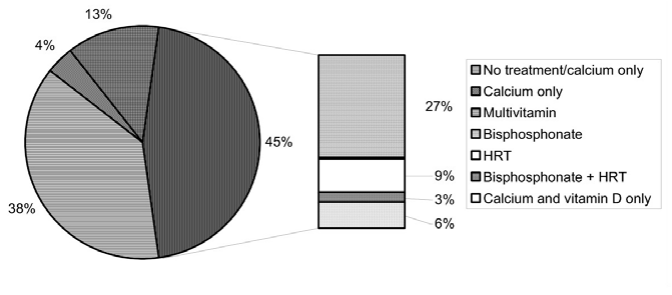
Osteoporosis therapy among subjects with a diagnosis of osteoporosis and/or fracture. (HRT = hormone replacement therapy) No treatment (n = 29) Calcium only (n = 3) Multivitamin (n = 10) Bisphosphonate (n = 21) HRT (n = 7) Bisphosphonate + HRT (n = 2) Calcium + vitamin D only (n = 5)

Specific therapeutic interventions for osteoporosis among subjects with a diagnosis of osteoporosis and/or fracture are shown in Figure 1. No subjects were using calcitonin or raloxifene. In total, only 35 subjects (45%) were receiving *at least *minimal treatment for osteoporosis (i.e. at least calcium with vitamin D). Only 30 subjects (39%) were receiving therapy with an approved prescription drug (bisphosphonate and/or hormone replacement therapy), with 5 subjects (6%) being treated only with calcium and vitamin D. Six subjects receiving prescription drug therapy were also taking calcium and vitamin D (not shown in Figure). Of the 42 subjects not receiving minimal treatment (i.e. drug therapy and/or calcium with vitamin D), 3 were taking calcium only, and 10 were taking a multivitamin that may have contained calcium and/or vitamin D. Bivariate comparisons of potential factors that may influence receipt of treatment are summarized in Table 3. A greater proportion of nonadherent subjects were observed in the group that did not receive treatment (50% *versus *31%). The proportion of subjects with lower education levels was also relatively higher among the non-treated group. None of the 5 men among the osteoporotic group were receiving treatment.

**Table 3:** Comparison of osteoporotic subjects by treatment status. (n = 77)

**Variable**	**No Treatment n = 42 % (n)**	**Treatment n = 35 % (n)**	**Fisher's exact p-value**
Age (≥ 85)	42.8 (18)	42.8 (15)	1.00
Sex (male)	11.9 (5)	0	0.06
Education			
(≥ High School)	38.1 (16)	54.3 (19)	0.18
Residence (seniors' lodge)	40.4 (17)	28.6 (10)	0.34
Cognitively impaired	23.8 (10)	22.8 (8)	0.60
Depression	14.3 (6)	14.3 (5)	0.75
Confined to wheelchair/bed	21.4 (9)	20.0 (7)	1.00
Fall(s)in past 90 days	31.0 (13)	22.9 (8)	0.45
On steroid medication	4.76 (2)	11.4 (4)	0.40
Nonadherent (with any prescribed medication)	50.0 (21)	31.4 (11)	0.11

## Discussion

Home care clients are expected to have relatively frequent contact with health care providers. Among those with a diagnosis of osteoporosis or prevalent fracture (n = 78), 30% had weekly contact with nursing staff, 42% had at least one emergency or emergent care visit or hospitalization in the past 3 months, and the majority (67%) had a recent (<6 months) review of their total medication regimen. Thus, more opportunity for intervention following fracture, and higher rates of treatment would be expected among this population. Although the proportion of home care clients receiving treatment was somewhat higher than reported in most previous studies (45% *versus *<39%, respectively) [5-8], [10-13], the majority of subjects with a diagnosis of osteoporosis or fracture indicative of osteoporosis were still not receiving even minimal therapy (i.e. calcium and vitamin D). Consistent with previous reports [14], we also found that men were not receiving therapy following fracture. In this home care sample, none of the 5 men with indication for osteoporosis treatment were receiving therapy. Three of these men had fractures at sights other than at the hip, where lower intervention rates were observed even for women. However, 2 of these men had a charted diagnosis of osteoporosis and were still not receiving treatment.

Our data also indicate that subjects who fracture at sites other than the hip may be less likely to receive treatment. While many of the fractures observed in this study (Table 2) were typical of osteoporotic fractures with respect to fracture site [2,4], our data are limited in that the MDS-HC does not include an assessment of the cause of the fracture. If some fractures were the result of trauma, our findings may overestimate the deficit in osteoporosis therapy. Conversely, the prevalence of osteoporosis may be underestimated in our study population, as the diagnosis was based on self-report information. The prevalence of osteoporosis in Canadian women aged 50+ years, based on BMD, has been estimated at 15% [20]. If diagnosis were based on BMD alone, a higher prevalence would be expected in the older population in this study. However, physicians' diagnoses of osteoporosis may derive from factors other than BMD, and our findings are consistent with previous work that suggests that many patients with osteoporosis are not receiving appropriate therapy.

We also examined several factors potentially associated with undertreatment. However, the small numbers of subjects with osteoporosis or prevalent fracture limit the interpretation of our findings. Although some trends are apparent, such as undertreatment of men and those with lower education, we cannot comment conclusively on these observations due to the limited sample size and cross-sectional nature of the study. Further evaluation of these factors utilizing a larger sample and appropriate multivariable analysis may provide further insight regarding the potential impact of specific determinants.

Speculation regarding reasons for the apparent undertreatment of osteoporosis has focused on physician oversight. However, several factors may play a role in the choice to initiate treatment. A recent study indicated that some physicians have concerns about the proven efficacy of osteoporosis treatment among older populations and may be exercising judgment with respect to minimizing polypharmacy in this population [21]. Further, physicians are not the only factor involved in decisions to initiate, and/or continue with therapy. In a study conducted by McKercher et al., physicians reported that the choice to initiate osteoporosis treatment was also dependent upon acceptance of the therapy by patients and/or family members [21]. Interviews with patients following fragility fracture also indicate that some patients choose not to use osteoporosis therapy [8,13] (e.g. concerns about side effects). The higher rate of nonadherence with medications among those not receiving therapy in this study, also suggests that lack of therapy may be due to patient choices. However, the assessment of adherence by self-report, as used in this study, only provided a measure of adherence with overall drug regimens, not specific medications. Collection of more detailed information on adherence with specific osteoporosis therapies in future studies may clarify this association.

Despite limitations, our findings highlight the growing concern that many patients with osteoporosis are not receiving appropriate therapeutic interventions. The lower treatment rate among subjects with fractures at sites other than the hip suggests that physicians may not be recognizing the probability of underlying osteoporosis. While the diagnosis of osteoporosis by BMD measurement has been widely publicized, recent clinical practice guidelines have emphasized the importance of a history of fragility fracture in the identification of patients with osteoporosis [3]. Our findings suggest that improving recognition of osteoporosis among older persons presenting with fractures may be an important educational objective for practicing physicians.

## Conclusions

Four previous studies conducted in Canada have examined osteoporosis diagnosis and treatment interventions following fracture. Our data confirm low treatment rates among patients with fracture, and also indicate that even patients with a documented diagnosis may not be receiving therapy. The reasons for lack of treatment of osteoporosis are not yet clear. The reports of patient and physician concerns regarding side effects and polypharmacy warrant further investigation, and suggest that nonpharmacologic interventions may be more acceptable in certain patient populations. Considering the complexity of issues involved in decisions to initiate and continue with treatment, future studies focusing more on evaluation of physician and patient awareness of osteoporosis and factors influencing treatment decisions are needed.

Evaluation of other patient-related factors, such as adherence and persistence with osteoporosis therapy, and their impact on health outcomes (e.g. fracture), are also relevant. Such studies may provide insight regarding specific interventions needed to reduce risk of morbidity and mortality associated with osteoporosis.

## Competing interests

Shelly Vik and Colleen Maxwell have no competing interests. David Hanley's competing interests include consultancies with, honoraria for speaking from, or involvement in research with, the following companies or organizations: Amgen, Astra-Zeneca, Aventis, the Dairy Farmers of Canada, Eli Lilly, Merck, Novartis, NPS Pharmaceuticals, Pfizer, Procter and Gamble, Roche, and Wyeth.

## Authors' contributions

Analysis of data, interpretation and the original draft were completed by Shelly Vik. Colleen Maxwell and David Hanley contributed to conception and interpretation, and provided critical evaluation of clinical and methodological content, and subsequent revisions.

## Pre-publication history

The pre-publication history for this paper can be accessed here:


